# Tumor suppressor let-7 acts as a key regulator for pluripotency gene expression in Muse cells

**DOI:** 10.1007/s00018-023-05089-9

**Published:** 2024-01-23

**Authors:** Gen Li, Shohei Wakao, Masaaki Kitada, Mari Dezawa

**Affiliations:** 1 Department of Stem Cell Biology and Histology, Tohoku University Graduate School of Medicine, 2-1 Seiryo-machi, Aoba-ku, Sendai, Miyagi 980-8575 Japan; 2 Department of Anatomy, Kansai Medical University School of Medicine, 2-5-1 Shin-machi, Hirakata, Osaka 573-1191 Japan

**Keywords:** Muse cells, Mesenchymal stem cells, Let-7, LIN28, PI3K-AKT pathway, MEK/ERK pathway

## Abstract

In embryonic stem cells (ESCs) and induced pluripotent stem cells (iPSCs), the expression of an RNA-binding pluripotency-relevant protein, LIN28, and the absence of its antagonist, the tumor-suppressor microRNA (miRNA) let-7, play a key role in maintaining pluripotency. Muse cells are non-tumorigenic pluripotent-like stem cells residing in the bone marrow, peripheral blood, and organ connective tissues as pluripotent surface marker SSEA-3(+). They express pluripotency genes, differentiate into triploblastic-lineage cells, and self-renew at the single cell level. Muse cells do not express LIN28 but do express let-7 at higher levels than in iPSCs. In Muse cells, we demonstrated that let-7 inhibited the PI3K-AKT pathway, leading to sustainable expression of the key pluripotency regulator KLF4 as well as its downstream genes, *POU5F1*, *SOX2*, and *NANOG*. Let-7 also suppressed proliferation and glycolysis by inhibiting the PI3K-AKT pathway, suggesting its involvement in non-tumorigenicity. Furthermore, the MEK/ERK pathway is not controlled by let-7 and may have a pivotal role in maintaining self-renewal and suppression of senescence. The system found in Muse cells, in which the tumor suppressor let-7, but not LIN28, tunes the expression of pluripotency genes, might be a rational cell system conferring both pluripotency-like properties and a low risk for tumorigenicity.

**Supplementary Information:**

The online version contains supplementary material available at 10.1007/s00018-023-05089-9.

## Introduction

The RNA-binding protein LIN28 and micro RNA (miRNA) lethal-7 (let-7) family members were first discovered in *Caenorhabditis elegans* (*C. elegans*) and are conserved across species [1–5]. In mammals, LIN28 and let-7 work as a mutually antagonistic system. LIN28 binds to the loop of precursor-let-7 (pre-let-7) to inhibit its maturation, and conversely, let-7 binds to the 3′ untranslated region (UTR) of *LIN28* to inhibit its translation [6, 7]. In this manner, the LIN28-let-7 axis widely controls many biologic processes, such as stem cell maintenance, development, differentiation, and cellular metabolism [8–11]. For example, LIN28 is highly expressed during early embryogenesis but its expression declines during development [12]. In mouse zygotes, LIN28 knockdown induces arrest between the 2- and 4-cell-stages, leading to a developmental failure at the morula and blastocyst stages, which suggests its importance in early development [13]. LIN28 and pluripotent factors POU5F1, SOX2, and NANOG are sufficient to convert human fibroblasts into induced pluripotent stem cells (iPSCs) [14], suggesting that LIN28 is a key regulator for controlling pluripotency. The LIN28-let-7 axis works in a seesaw manner to maintain the balance between self-renewal and differentiation in pluripotent stem cells (PSCs) such as embryonic stem cells (ESCs) and iPSCs; the expression of LIN28 is high in undifferentiated ESCs and iPSCs and decreases during cell differentiation [4, 15, 16], while expression of the let-7 family, which is not observed in undifferentiated ESCs and iPSCs, increases during differentiation.

After birth, most somatic cells lose the expression of LIN28. The recurrence of LIN28 expression, however, is observed in many human cancers, such as breast-, colon-, liver-, and ovarian cancers [17]. LIN28 is considered a marker for cancer stem cells and could be a target for anticancer therapies [18–20]. Therefore, LIN28 is considered an oncogene. In contrast, let-7 is downregulated in many cancer cells [21–23], and forced expression of let-7 induces a slowdown of tumor growth [24]. Therefore, let-7 is considered a tumor suppressor miRNA. Thus, the high expression of let-7 in the majority of somatic cells might be a strategy to decrease tumorigenic risk.

Multilineage differentiating stress-enduring (Muse) cells are endogenous reparative pluripotent-like stem cells that reside in the bone marrow, peripheral blood, and organ connective tissue as cells positive for a pluripotency surface marker, stage-specific embryonic antigen (SSEA)-3 [25–28]. Muse cells are also collectible as several percent of SSEA-3(+) cells from cultured mesenchymal stromal cells (MSCs) and fibroblasts. Muse cells express other pluripotency markers, including NANOG, POU5F1, and SOX2, at moderate levels compared with ESCs and iPSCs [26, 29]. They can generate endodermal-, mesodermal-, and ectodermal-lineage cells and self-renew at the single cell level; they also exhibit stress tolerance due to a high capacity for sensing and repairing DNA damage [26, 30, 31]. Unlike ESCs and iPSCs, however, Muse cells are non-tumorigenic, consistent with the fact that they are endogenous to the body; express telomerase at a low level, comparable to that in somatic cells such as fibroblasts; and do not form teratomas after transplantation in vivo [26]. Circulating endogenous Muse cells and intravenously administered exogenous Muse cells both selectively home to damaged tissue by sensing sphingosine-1-phosphate, a damage signal produced by damaged tissue; phagocytose apoptotic differentiated cell fragments to receive differentiation machineries such as transcription factors; initiate differentiation into the same cell type as the phagocytosed cells in a short time period; and repair the tissue by replacing damaged cells [28, 32–36]. As demonstrated in a rabbit acute myocardial infarction model, allogeneic Muse cells can escape host immunologic attack and survive as functional cells in the host tissue for more than half a year without immunosuppression. The immune privilege of Muse cells is partly explained by the expression of human leukocyte antigen (HLA)-G, which is expressed in extravillous trophoblast cells in the placenta and plays an important role in immune tolerance during pregnancy [35]. Clinical trials are currently being conducted for stroke, acute myocardial infarction, epidermolysis bullosa, spinal cord injury, amyotrophic lateral sclerosis, and COVID19-acute respiratory distress syndrome using intravenous injections of human clinical-grade Muse cells without HLA-matching or immunosuppression. The safety and effectiveness of Muse cells have been reported in clinical trials of epidermolysis bullosa, acute myocardial infarction, and subacute ischemic stroke [37–39].

To clarify the mechanisms underlying how Muse cells maintain their pluripotency-like properties without being tumorigenic, we evaluated the LIN28-let-7 axis in Muse cells. We found that Muse cells did not express LIN28 but expressed let-7 at higher levels than iPSCs. We also explored the mechanisms by which let-7 acts as a key regulator of pluripotency gene expression and its involvement in suppressing proliferation and glycolysis in Muse cells.

## Results

### Expression of LIN28 and let-7 in Muse cells

**Fig. 1:**
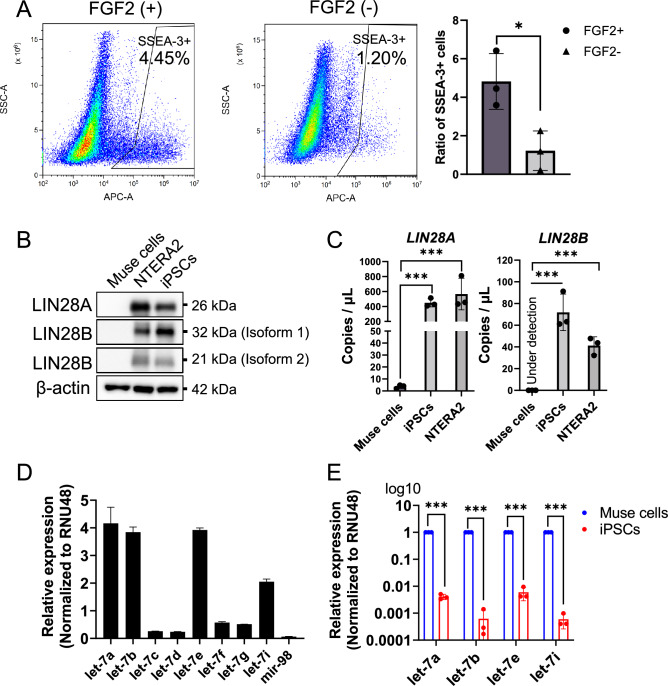
Muse cells express let-7 but not LIN28. **A** The ratio of the Muse cell population, which was cultured with or without FGF2 treatment (*n* = 3). **B** Western blot of LIN28A/B in Muse cells, NTERA2, and iPSCs. β-Actin was used as an endogenous control. **C** Expression of *LIN28A* and *LIN28B* in Muse cells, NTERA2, and iPSCs in ddPCR. Data are shown as DNA copies/μL (all, *n* = 3). **D** Expression of let-7 subtypes in BM-Muse cells in qPCR (all *n* = 3). RNU48 was used as an endogenous control. **E** qPCR of let-7a, -7b, -7e, and -7i in Muse cells and iPSCs (*n* = 3). RNU48 was used as an endogenous control. A log10 scale was used for the y-axis

We isolated SSEA-3(+)-Muse cells from human bone marrow (BM)-MSCs and normal human dermal fibroblasts (NHDFs) as reported previously (Supplemental Fig. S1A and B) [26, 40]. When the BM-MSCs were cultured with fibroblast growth factor 2 (FGF2) for 2 population doubling levels (PDLs), the ratio of the Muse cell population among MSCs was 4.82% on average from 3 replicates, similar to previous reports [40] (Fig. 1A). When the BM-MSCs were cultured without FGF2, the Muse cell ratio decreased to 1.22% on average from 3 replicates (Fig. 1A). Therefore, FGF2 is indispensable for maintaining Muse cells in BM-MSC populations.

In Western blotting, both LIN28A and LIN28B were under the detection limit in Muse cells, while they were detectable in the human teratoma cell line NTERA2 and human iPSCs (Fig. 1B). In droplet digital PCR (ddPCR), the expression of *LIN28A* in Muse cells was significantly lower than that in iPSCs and NTERA2; the expression of *LIN28B* was under the detection limit in Muse cells, but clearly detectable in iPSCs and NTERA2 (Fig. 1C). Quantitative polymerase chain reaction (qPCR) showed that the major let-7 subtypes expressed in Muse cells were let-7a, -7b, -7e, and -7i among the 9 subtypes (Fig. 1D). The expression of let-7a, -7b, -7e, and -7i was 100–1000 times higher in Muse cells than in iPSCs (Fig. 1E). These findings indicated that, in contrast to iPSCs, Muse cells basically express let-7 but not LIN28A/B. Muse cells isolated from NHDFs showed similar trends of LIN28A/B and let-7 expression (Supplemental Fig. S1C–F). We used BM-MSC–derived Muse cells in the following experiments and focused on the let-7a, let-7b, let-7e, and let-7i subtypes.

### Gene expression profile in Muse cells after let-7 knockdown

**Fig. 2:**
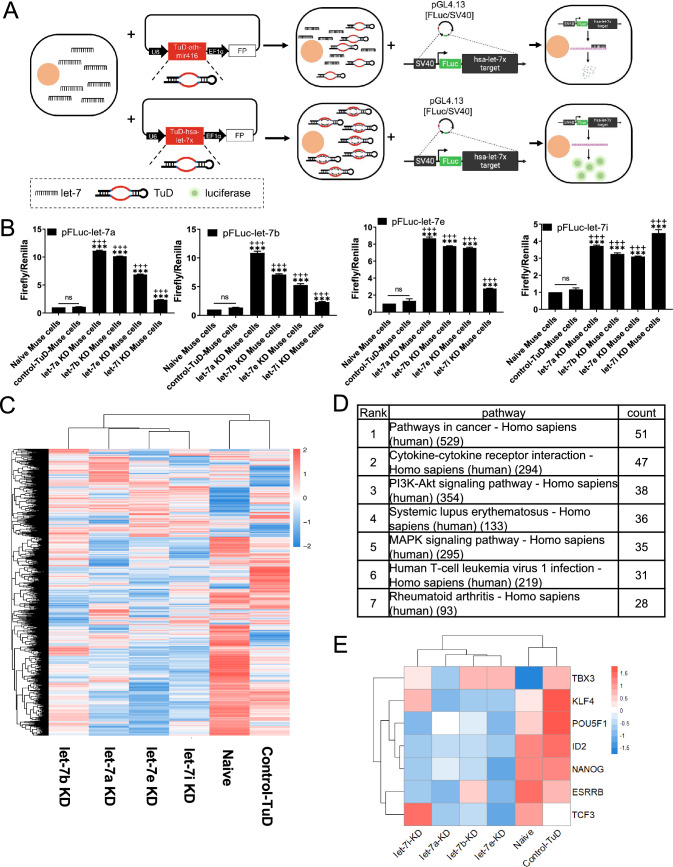
let-7 TuD knockdown and bioinformatic analysis by microarray. **A** Experimental design of TuD-based let-7 KD and evaluation of let-7 KD by luciferase assay. FP (Fluorescent protein): either EGFP or mCherry, used as an indicator for transfected cells. FLuc: firefly luciferase. Let-7x represents let-7a, -7b, -7e, or -7i. **B** Luciferase assay for let-7 KD. pFLuc-let-7a, -7b, -7e, and -7i: pGL4.13 with firefly luciferase and let-7a, -7b, -7e, or -7i target sequence inserted in the 3′UTR of firefly luciferase. ***p < 0.001 versus naïve Muse cells; ^+++^*p* < 0.001 versus control-TuD-Muse cells; ns: no significant difference. **C** Microarray heatmap of let-7a-KD, -7b-KD, -7e-KD, -7i-KD, naïve and control-TuD Muse cells. **D** KEGG pathway analysis showing the top 7 changed pathways after let-7 KD. The number in the pathway column indicates the total factor number in the pathway, and the count indicates the number of changed factors after let-7 KD. Control-TuD-Muse cells were set as the control group. Let-7a-KD, let-7b-KD, let-7e-KD, and let-7i-KD Muse cells were combined as the experimental group. **E** Heatmap showing some of the changed pluripotency-relevant genes after let-7 KD. Red: upregulated. Blue: downregulated. White: no change

A sequence-specific miRNA inhibition system, tough decoy (TuD) [41], was constructed and introduced into Muse cells by lentivirus for the loss of function of let-7 (Figs. 2A and S2A). We performed a luciferase assay to confirm the activity of TuD-based let-7 knockdown (KD). We first constructed 4 luciferase-expression plasmids with the target sequences of let-7a, -7b, -7e, and -7i inserted into the 3′UTR of firefly luciferase: pFluc-let-7a, pFluc-let-7b, pFluc-let-7e, and pFluc-let-7i, respectively. We also prepared 6 types of Muse cells; non-transfected naïve Muse cells, negative control-TuD-ath-mir416 introduced Muse cells (control-TuD-Muse cells); and let-7a-KD, let-7b-KD, let-7e-KD, and let-7i-KD Muse cells. The plasmids were each introduced into the 6 types of Muse cells (Fig. S2B). We measured the luciferase intensity. When the pFluc-let-7a plasmid was transfected into each type of Muse cell, the luminescence signal of the luciferase was significantly increased in the let-7a-KD-, let-7b-KD, let-7e-KD, and let-7i-KD-Muse cells compared with naïve and control-TuD-Muse cells. A similar tendency was confirmed for pFluc-let-7b, -7e, and -7i (Fig. 2B). Thus, the TuD-let-7 system effectively knocked down let-7a, -7b, -7e, and -7i expression in Muse cells, although the inhibition specificity was not high among these 4 subtypes, probably due to sequence similarities among the subtypes (Fig. S2C). Let-7a-KD-Muse cells were collected from BM-MSCs after 3–4 PDLs after introducing the TuD-let-7a lentivirus. TUNEL staining results showed that the apoptotic cell ratio was not largely increased by transfecting Muse cells with the TuD-let-7a lentivirus (Fig. S2D). Similar trends were confirmed with the TuD-let-7b, -let-7e, and -let-7i lentivirus transfected cells (data not shown).

A DNA microarray analysis was conducted to assess the transcriptome changes after let-7-KD in Muse cells. Figure 2C shows a cluster heatmap illustrating that the gene expression pattern was changed in let-7a-KD-, 7b-KD-, 7e-KD-, and 7i-KD-Muse cells compared with naïve- and control-TuD-Muse cells. Because the TuD inhibition system did not specifically downregulate each of the 4 let-7 subtypes in Muse cells, we combined the microarray gene profiles of let-7a-, let-7b-, let-7e-, and let-7i-KD Muse cells into a single group and performed the Kyoto Encyclopedia of Genes and Genomes (KEGG, https://www.genome.jp/kegg/) pathway frequency analysis for comparison with the control-TuD-Muse cell group. The KEGG pathway frequency analysis revealed the top 7 changed pathways between the control-TuD- and let-7 (-7a, -7b, -7e, and -7i) groups, which included cancer, cytokine-cytokine receptor interaction, and the PI3K-AKT and MEK/ERK signaling pathways (Fig. 2D). Interestingly, let-7-KD (-7a, -7b, -7e, and -7i)-Muse cells exhibited the downregulation of pluripotency-related genes such as *KLF4*, *POU5F1*, *ID2*, *NANOG*, and *ESRRB* (Fig. 2E), and the upregulation of cell cycle-relevant genes such as Cyclin D2 (CCND2), cell division cycle 25A (CDC25A), origin recognition complex, minichromosome maintenance proteins, and retinoblastoma protein in comparison with naïve- and control-TuD-Muse cells (Supplemental Fig. S2E). In addition, *P53* was downregulated by let-7 KD (Supplemental Fig. S2E).

As the expression of let-7a was the highest among the let-7 subtypes in BM-Muse cells (Fig. 1D) and the knockdown effect of the TuD structure was not specific (Fig. 2B), the TuD-let-7a KD system was applied in the following experiments. To predict let-7a targets, we utilized the Encyclopedia of RNA Interactomes (ENCORI, http://starbase.sysu.edu.cn/), a platform that provides information on miRNA targets collated from 7 databases (i.e., PITA, RNA22, miRmap, microT, miRnada, PicTar, and TargetScan). Genes predicted as let-7a target genes by more than 4 databases were screened, and those screened genes were then further analyzed using protein–protein interactions analysis with String (https://string-db.org/) [42].

**Fig. 3:**
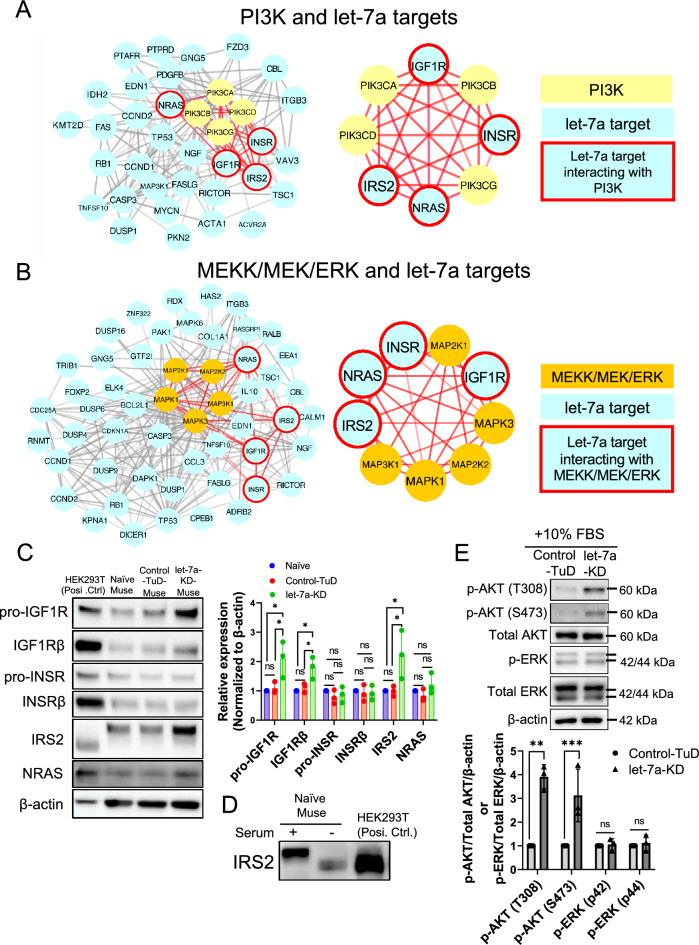
Prediction and confirmation of let-7 targets in Muse cells. **A** Protein–protein interactions of the putative let-7a targets and PI3K. Blue: let-7a targets predicted by ENCORI; Yellow: PI3K isoforms; Blue with red frame: putative let-7a targets interacting with PI3K. **B** Protein–protein interactions of the putative let-7a targets and MEKK/MEK/ERK. Blue: let-7a targets predicted by ENCORI; Orange: isoforms of MEKK, MEK, or ERK; Blue with red frame: putative let-7a targets interacting with MEKK, MEK, or ERK. **C** Western blot of predicted let-7a targets in Muse cells (*n* = 3). Posi. Ctrl. (Positive control): HEK293T. Signals of each predicted target were normalized by β-actin. **D** Western blot of IRS2 under serum or serum-free conditions. Posi. Ctrl. (Positive control): HEK293T. **E** Western blot of p-AKT and p-ERK in control-TuD- and let-7a-KD-Muse cells under 10% FBS culture condition (*n* = 3). β-Actin was used as endogenous control. p-AKT/total AKT/β-actin: The p-AKT intensity was divided by that of total AKT, and then further divided by that of β-actin to determine the quantitative value. Similarly, the p-ERK intensity was divided by that of total ERK, and then further divided by that of β-actin: P42 and p44 were calculated separately

We focused on protein–protein interactions between the predicted let-7a targets and PI3K (PIK3CA, PIK3CB, PIK3CD, and PIK3CG) or MEKK/MEK/ERK (MAPK1, MAPK3, MAP2K1, MAP2K2, and MAP3K1) because these 2 pathways were listed in the top 7 pathways that were different between the control-TuD- and let-7 groups (Fig. 2D). Among the predicted let-7a target genes, insulin receptor substrate 2 (IRS2), insulin like growth factor 1 receptor (IGF1R), insulin receptor (INSR), and neuroblastoma RAS viral oncogene homolog (NRAS) were predicted to interact with both the MEK/ERK and PI3K-AKT signaling pathways (Fig. 3A and B). We conducted Western blot analyses to examine the expression of the pro-form of IGF1R (pro-IGF1R), the IGF1R beta subunit (IGF1Rβ), the pro-form of INSR (pro-INSR), the INSRβ subunit (INSRβ), IRS2, and NRAS. Pro-IGF1R, IGF1Rβ, and IRS2 were significantly increased in let-7a-KD-Muse cells compared with naïve- and control-TuD-Muse cells (Fig. 3C), suggesting that IGF1R and IRS2 are targets of let-7a in Muse cells. NRAS, pro-INSR, and INSRβ expression, however, did not largely change among the naïve-, control-TuD-, and let-7a-KD-Muse cells (Fig. 3C). The molecular sizes of the Western blot signals for IRS2 differed between Muse cells and the positive control-HEK293T (Fig. 3C). This difference was considered to be the phosphorylation of IRS2 in naïve-, control-TuD-, and let-7a-KD-Muse cells in the presence of serum. We therefore used a serum-free medium to culture the Muse cells to eliminate the influence of phosphorylation on the molecular weight of IRS2 and found that the size of IRS2 in Muse cells was the same as that observed in HEK293T (Fig. 3D).

Activation of PI3K and its downstream cascade converge on phosphorylated AKT (p-AKT), indicating that PI3K is downstream of IGF1R/IRS2 and AKT is the critical molecule in the PI3K pathway [43]. In addition, IGF1R and IRS2 are reported to be let-7 targets [44, 45]. Thus, we compared the expression of p-AKT (T308), p-AKT (S473), and p-ERK (p42/p44) between control-TuD and let-7a-KD-Muse cells. In Muse cells, let-7a KD upregulated the expression of both p-AKT (T308) and p-AKT (S473), but not p-ERK (p42/p44) (Fig. 3E). Thus, let-7 inhibited the PI3K-AKT pathway by inhibiting IGF1R and IRS2, but had a limited influence on the MEK/ERK pathway.

### Effect of let-7 on PI3K-AKT and crosstalk between the MEK/ERK and PI3K-AKT pathways

**Fig. 4:**
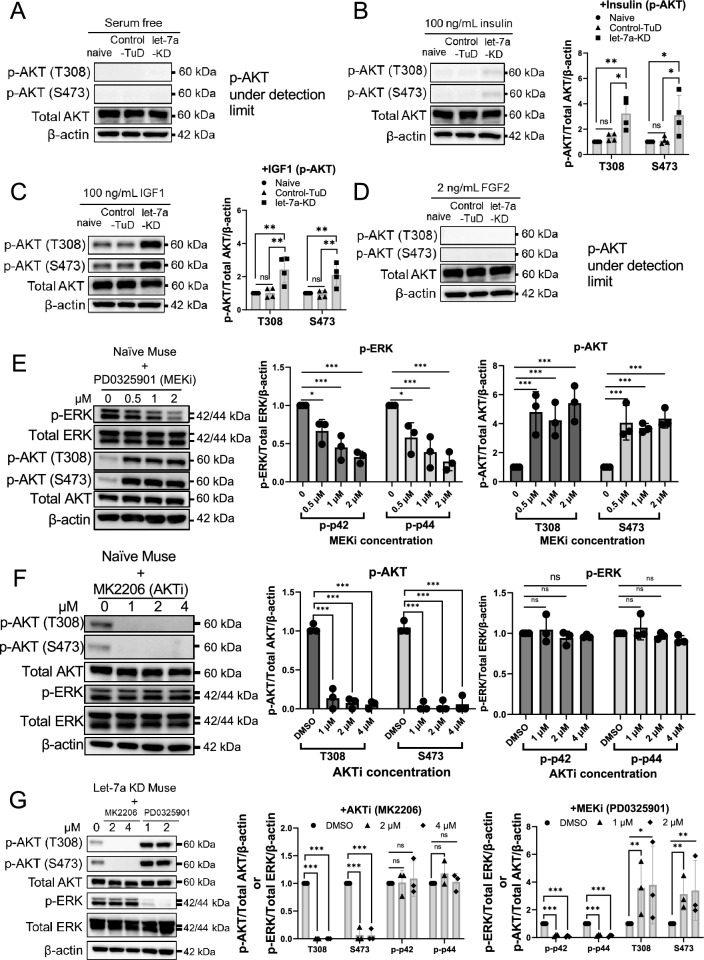
Effect of let-7 on PI3K-AKT and crosstalk between the MEK/ERK and PI3K-AKT. **A**–**D** Serum starvation eliminated AKT phosphorylated sites in naïve, control-TuD-, and let-7-KD-Muse cells. Adding insulin (100 ng/mL) or IGF1 (100 ng/mL) phosphorylated AKT at T308 and S473 sites, and p-AKT increased more in let-7-KD Muse cells than in naïve and control-TuD-Muse cells (all *n* = 4). Addition of 2 ng/mL FGF2 did not change the p-AKT level. **E** In naïve Muse cells, MEKi (PD0325901) inhibited p-ERK (p-p42/p-p44) in a dose-dependent manner. Inhibition of p-ERK increased the level of p-AKT (T308) and p-AKT (S473) (all, *n* = 3). **F** In naïve Muse cells, AKTi (MK2206) inhibited p-AKT (T308) and p-AKT (S473), but the expression of p-ERK (p-p42/p-p44) was not affected (all, *n* = 3). **G** In let-7a-KD Muse cells, the addition of AKTi (MK2206) and MEKi (PD0325901) inhibited p-AKT and p-ERK, respectively. MEKi increased p-AKT, but AKTi did not upregulate p-ERK. β-Actin was used as endogenous control. p-AKT/total AKT/β-actin: the p-AKT intensity was divided by that of total AKT, and then further divided by that of β-actin to determine the quantitative value. Similarly, the p-ERK intensity was divided by that of total ERK, and then further divided by that of β-actin: P42 and p44 were calculated separately

We examined how let-7-KD affected the PI3K-AKT pathway in Muse cells. To confirm activation of the PI3K pathway, we cultured naïve-, control-TuD-, and let-7a-KD-Muse cells for 8 h under serum-free conditions to remove the influence of phosphorylation by the growth factors contained in the serum. In the serum-free conditions, p-AKT (T308) and p-AKT (S473) were not detected in any of the 3 types of Muse cells (i.e., naïve, control-TuD, and let-7a-KD) on Western blots (Fig. 4A). After adding 100 ng/mL insulin to the serum-free medium for 15 min, the p-AKT (T308) and p-AKT (S473) levels increased in all 3 types of Muse cells. The highest increase of p-AKT (T308) and p-AKT (S473) was in let-7-KD-Muse cells compared with naïve- and control-TuD-Muse cells (Fig. 4B). A similar trend was observed after the addition of 100 ng/mL IGF1 to the serum-free culture medium for 15 min. Notably, the increased p-AKT (T308) and p-AKT (S473) levels were higher in 100 ng/mL IGF1-treated naïve, control-TuD, and let-7a-KD Muse cells than in 100 ng/mL insulin-treated cells (Fig. 4B and C).

FGF2 is important for maintaining Muse cells, as suggested in Fig. 1A. Therefore, the effect of FGF2 on the PI3K-AKT pathway was examined. The increased AKT phosphorylation, however, remained under the detection limit when 2 ng/mL FGF2 was supplied to the serum-free culture medium for 15 min (Fig. 4D). Thus, let-7a-KD activated the PI3K-AKT pathway in the presence of insulin or IGF1, but not FGF2, in Muse cells.

The interaction between the MEK/ERK and PI3K-AKT pathways was investigated in naïve Muse cells. PD0325901, a MEK inhibitor (MEKi), induced a dose-dependent decrease in p-ERK. The p-AKT (T308) and p-AKT (S473) levels, however, were both upregulated in naïve Muse cells (Fig. 4E). Treating Muse cells with MK2206, an AKT inhibitor (AKTi), inhibited the phosphorylation of AKT at T308 and S473, but did not affect p-ERK (Fig. 4F). These results suggested that, in naïve Muse cells, MEK/ERK inhibited the PI3K-AKT pathway by inhibiting the phosphorylation of AKT, while AKT inhibition did not affect the phosphorylation of ERK.

In let-7a-KD-Muse cells, MEKi also upregulated p-AKT (T308) and p-AKT (S473), while AKTi-induced inhibition of p-AKT did not affect p-ERK expression, similar to the findings in naïve Muse cells (Fig. 4G). These results suggested that, although let-7a KD changed the transcriptome of Muse cells, the crosstalk between the PI3K-AKT and MEK/ERK pathways was not largely affected.

### Let-7 KD decreased the expression of pluripotency genes

**Fig. 5:**
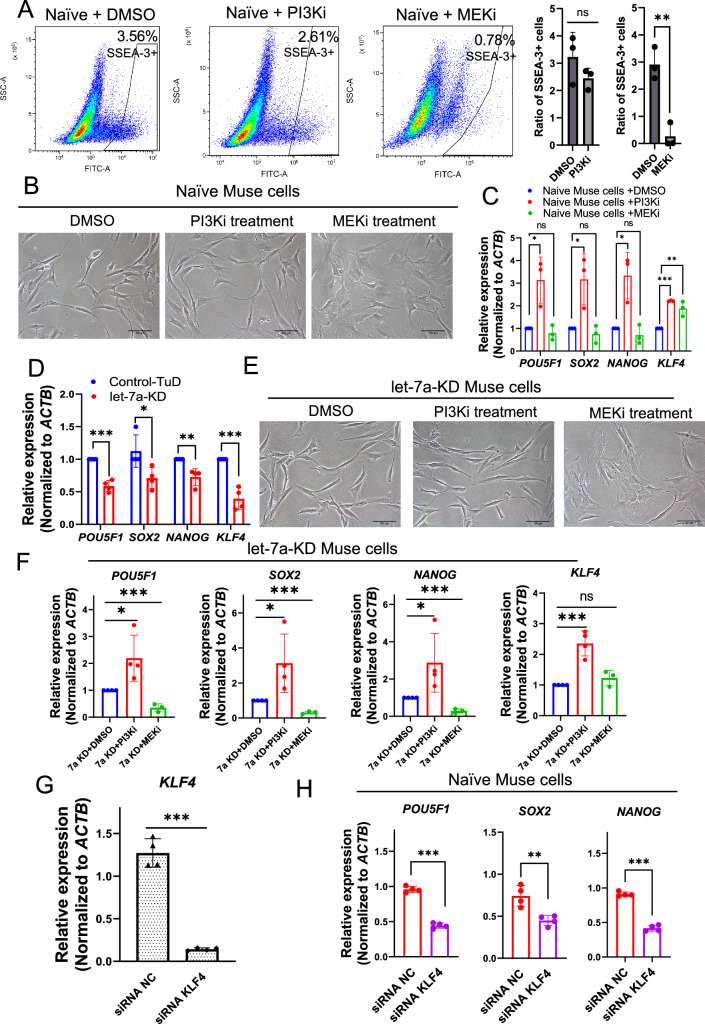
let-7 promoted the expression of pluripotency genes. **A** Flow cytometry analysis of Muse cells in DMSO-, PI3Ki-, and MEKi-treated hMSCs. MEKi decreased the ratio of Muse cells, but PI3Ki showed no significant effect (*n* = 3). **B** Cell morphology of naïve Muse cells 24 h after DMSO, PI3Ki, and MEKi treatment. Scale bar: 100 µm. **C** Comparison of pluripotency gene expression among DMSO-, PI3Ki-, and MEKi-treated naïve Muse cells by qPCR (all *n* = 3). **D** Expression of pluripotency genes between control-TuD and let-7a-KD Muse cells in qPCR (all *n* = 4). **E** Cell morphology of let-7a-KD Muse cells 24 h after DMSO, PI3Ki, and MEKi treatment. Scale bar: 100 µm. **F** qPCR of *POU5F1*, *SOX2*, and *NANOG* in DMSO- (*n* = 4), PI3Ki- (*n* = 4), and MEKi-treated (*n* = 3) let-7a-KD Muse cells. **G** qPCR of *KLF4* in negative control (NC) siRNA and KLF4 knockdown siRNA (*n* = 4). **H** qPCR of *POU5F1*, *SOX2*, and *NANOG* in negative control (NC) siRNA and KLF4-knockdown siRNA-introduced naïve Muse cells (*n* = 4). PI3Ki: LY294002. MEKi: PD0325901. ACTB was used as an endogenous control in all the qPCR experiments

We treated BM-MSCs with LY294002, a PI3K inhibitor (PI3Ki), and MEKi for 2 PDLs, and examined the Muse cell population ratio. The results of 3 replicates showed that, compared with the DMSO-treated group, PI3Ki treatment did not largely affect the Muse cell ratio, while MEKi significantly reduced the Muse cell ratio to less than 1% (Fig. 5A). These results suggested that the MEK/ERK pathway, but not the PI3K-AKT pathway, has a pivotal role in maintaining Muse cells.

We then examined how *POU5F1*, *SOX2*, *NANOG*, and *KLF4* were affected by PI3Ki and MEKi treatment in naïve Muse cells. The morphology of naïve Muse cells did not remarkably change after PI3Ki treatment for 24 h, while the morphology of MEKi-treated Muse cells became flattened (Fig. 5B). PI3Ki treatment induced a significantly higher (2–4fold) expression of *POU5F1*, *SOX2*, *NANOG*, and *KLF4* in naïve Muse cells compared with DMSO-treated naïve Muse cells (Fig. 5C). MEKi incubation for 24 h, however, did not change the *POU5F1*, *SOX2*, and *NANOG* expression compared with that in DMSO-treated naïve Muse cells, but increased the expression of *KLF4* by ~ 2 times (Fig. 5C).

Following differentiation of ESCs, pluripotency gene expression decreases and let-7 expression increases [46, 47]. In contrast to ESCs, let-7a-KD significantly decreased *POU5F1*, *SOX2*, *NANOG*, and *KLF4* expression in Muse cells compared with control-TuD-Muse cells (Fig. 5D), consistent with the microarray data (Fig. 2E). We then treated TuD-let-7a-KD Muse cells with PI3Ki or MEKi for 24 h. PI3Ki-treated let-7a-KD Muse cells did not show clear changes in cell morphology. In contrast, the morphology of MEKi-treated let-7a-KD Muse cells became flattened (Fig. 5E). The qPCR results showed that treating let-7a-KD Muse cells with PI3Ki for 24 h reversed the effects of let-7a-KD; expression of *POU5F1*, *SOX2*, *NANOG*, and *KLF4* was significantly elevated compared with that in control (DMSO supplied-let-7a-KD) Muse cells (Fig. 5F). This finding suggested that let-7 controlled the expression of these genes through the PI3K-AKT pathway.

MEKi treatment of let-7a-KD for 24 h significantly suppressed the expression of *POU5F1*, *SOX2*, and *NANOG* compared to DMSO-treated let-7a-KD Muse cells (Fig. 5F). Due to the morphology changes after MEKi treatment, we considered that the decrease in *POU5F1*, *SOX2*, and *NANOG* expression might relate to cell senescence or apoptosis. To investigate this, we cultured control-TuD- and let-7a-KD-Muse cells with MEKi for 5 days. Flow cytometry analysis of SPiDER β-gal staining showed that MEKi treatment nonsignificantly increased senescence-associated beta-galactosidase (SA-βgal) intensity ~ 1.5 times more in control-TuD Muse cells than in DMSO-treated Muse cells, whereas it significantly (~ twofold) increased SA-βgal intensity in let-7a-KD Muse cells compared with DMSO-treated Muse cells on days 1, 3, and 5 (Supplemental Fig. S3A). PI3Ki treatment did not increase the fluorescence intensity in either the control-TuD- or let-7a-KD-Muse cells (Supplemental Fig. S3B). The flow cytometry results of AnnexinV-FITC staining showed that neither PI3Ki nor MEKi induced severe apoptosis compared with the UV-treated positive control (Supplemental Fig. S3C). These findings suggested that MEKi induced cell senescence rather than apoptosis in let-7a-KD Muse cells and that let-7a inhibits cellular senescence when the MEK/ERK pathway is blocked.

KLF4 controls the expression of SOX2 and NANOG in ESCs [48–50]. To examine whether KLF4 regulates the expression of *POU5F1*, *SOX2*, and *NANOG* in Muse cells, *KLF4* KD was conducted using small interfering RNA (siRNA). Western blotting showed a sharp decrease in KLF4 in Muse cells at 2 and 3 days after transfection of 3 different siRNAs (siRNA #1, siRNA #2, and siRNA #3; Supplemental Fig. S3D). When the 3 siRNAs for *KLF4* were mixed, qPCR confirmed the statistically significant suppression of *KLF4* on day 3 after siRNA transfection (Fig. 5G). Consequently, *POU5F1* (*p* < 0.001), *SOX2* (*p* < 0.01), and *NANOG* (*p* < 0.001) were suppressed in KLF4-KD-Muse cells compared with siRNA-negative control-introduced Muse cells (Fig. 5H). These findings suggested that KLF4 positively regulates *POU5F1*, *SOX2*, and *NANOG* expression in Muse cells.

### Overexpression of mature let-7 was not feasible in Muse cells

We attempted to overexpress mature let-7a, let-7b, let-7e, and let-7i in Muse cells by introducing pre-let-7-lentivirus (Supplemental Fig. S4A). Mature let-7a is generated from 3 kinds of pre-let-7a (Roush et al., 2008). We first compared their expression levels in naïve Muse cells. qPCR results showed that pre-let-7a-3 was dominantly expressed among the three pre-let-7a forms, corresponding to ~ 7 times and ~ 2 times higher than pre-let-7a-1 and pre-let-7a-2, respectively (Supplemental Fig. S4B). Therefore, we selected pre-let-7a-3 for the let-7 overexpression experiment. The pre-let-7a-3 was confirmed to be overexpressed by more than ~ 15 times compared with empty vector-transfected Muse cells (Supplemental Fig. S4C). While mature let-7a was overexpressed at an average of only 1.08 times higher than that in empty vector-transfected Muse cells after culturing for 5 PDLs, however, the increase was statistically significant (Supplemental Fig. S4C). In addition, lentiviral transfection with pre-let-7b and pre-let-7e failed to overexpress mature let-7b and let-7e in naïve Muse cells (Supplemental Fig. S4C). Both pre-let-7a-3 and mature let-7a were largely overexpressed in NTERA2 cells, demonstrating that the overexpression system worked well in NTERA2 cells but not in Muse cells (Supplemental Fig. S4D).

Notably, mature let-7i was significantly overexpressed (1.5–2 times higher than that in empty vector-transfected Muse cells) (Supplemental Fig. S4C). Luciferase assay confirmed that let-7i overexpression inhibited the expression of firefly luciferase compared with that in pGL4.13-empty vector-transfected cells (Supplemental Fig. S4E). We then examined the influence of let-7i overexpression on pluripotency gene expression. The expression of *POU5F1*, *SOX2*, *NANOG*, and *KLF4* did not significantly change in the let-7i overexpressing Muse cells compared with empty vector-transfected cells (Supplemental Fig. S4F). One possible explanation is that the effect of the let-7 family members on positive regulation of pluripotency genes had already reached a plateau prior to their overexpression.

### Let-7a KD accelerated Muse cell proliferation without activation of telomerase

**Fig. 6:**
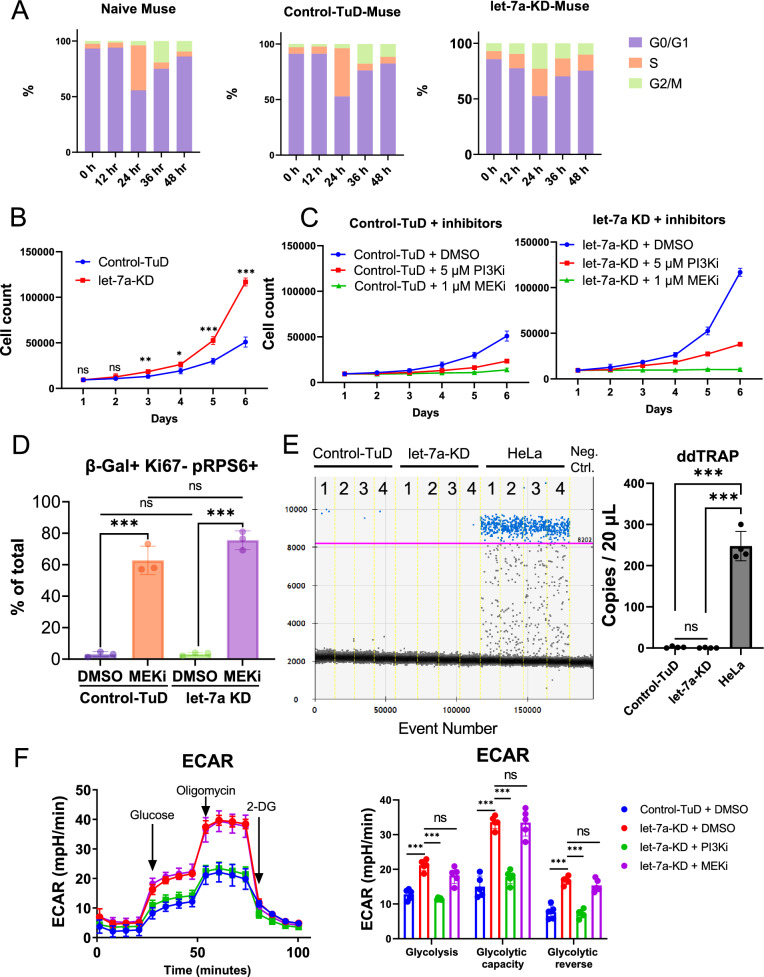
The influence of let-7 on cell proliferation and glycolysis. **A** Flow cytometry analysis of cell cycle variation among naïve, control-TuD-, and let-7a-KD-Muse cells by PI staining for 48 h. **B** Cell proliferation assay in control-TuD and let-7a-KD Muse cells by cell count (*n* = 4). **C** Cell proliferation assay in control -TuD and let-7a-KD Muse cells under the presence of DMSO, 5 μM PI3Ki, and 1 μM MEKi by cell count (all *n* = 4). **D** The percent of β-gal(+)/pRPS6(+)/Ki67(−) cells, representing senescent cells, to the total cell population was shown (*n* = 3). **E** Comparison of telomerase activity by ddTRAP among control-TuD, let-7a-KD-Muse cells, and HeLa cells (*n* = 4). HeLa cells were used as a positive control. No template control during the extension reaction was used as a negative control. Neg. ctrl.: negative control. The threshold was set at 8202 (purple line). **F** Comparison of ECAR among DMSO-treated control-TuD and let-7a-KD-Muse cells in the presence of DMSO, PI3Ki, and MEKi (*n* = 5). PI3Ki: LY294002. MEKi: PD0325901. ECAR: extracellular acidification rate. 2-DG: 2-deoxy-D-glucose

Cell cycle analysis by flow cytometry revealed that the percentage of naïve-Muse cells at the G0/G1, S, and G2/M phase was 93%, 4.4%, and 2.5% at 0 h, respectively (Fig. 6A). After seeding the cells on culture dishes, naïve Muse cells started entering S phase at 24 h. Control-TuD-Muse cells showed a similar trend. In let-7a-KD-Muse cells, the G0/G1, S, and G2/M percentages were similar to those in naïve- and control-TuD-Muse cells at 0 h. The let-7a-KD-Muse cells, however, started entering into S phase after 12 h, which was earlier than that of the naïve- and control-TuD-Muse cells (Fig. 6A). The proliferation speed of the control-TuD- and let-7a-KD-Muse cells was examined for 6 days. Let-7a-KD-Muse cells had higher proliferation activity than control-TuD-Muse cells starting on day 3 (*p* < 0.01), and the growth rate was 2.3 times higher in let-7a-KD-Muse cells than in control-TuD-Muse cells (*p* < 0.001) on day 6 (Fig. 6B). These results demonstrated that let-7a KD accelerated the cell cycle and proliferation speed in Muse cells.

The effect of PI3Ki and MEKi on the proliferation of let-7a-KD-Muse cells was also examined. In both control-TuD- and let-7a-KD-Muse cells, PI3Ki suppressed proliferation activity compared with those of DMSO treated-Muse cells, while MEKi induced growth arrest in both types of Muse cells (Fig. 6C). Senescent cells can be identified as cells positive for β-Gal and pRPS6 and negative for Ki67 [51, 52]. The triple staining for β-Gal, pRPS6 and Ki67 revealed that MEKi treatment increased the ratio of β-Gal(+)/pRPS6(+)/Ki67(-) in both control-TuD and let-7a KD cells on day 5 compared to their DMSO-treated counterparts (Supplemental Fig. S5A and Fig. 6D). However, there was no significant difference between MEKi-treated control-TuD and let-7a KD Muse cells. These results demonstrated that PI3Ki suppressed proliferation activity, and MEKi induced senescence in Muse cells.

Telomerase activity is one of the indicators that reflect the replicative immortality of cancer cells [53]. Droplet digital telomere repeat amplification protocol (ddTRAP) demonstrated that the telomerase activity was under the detection limit in both control-TuD and let-7a-KD Muse cells but was significantly higher in HeLa cells (Fig. 6E), suggesting that let-7a KD did not clearly evoke the activation of telomerase in Muse cells.

### Let-7 inhibited glycolysis through the PI3K-AKT pathway in Muse cells

To investigate the effect of let-7a on cellular metabolism in Muse cells, we measured the extracellular acidification rate (ECAR), an indicator of glycolysis. In let-7a-KD-Muse cells, glycolysis, glycolytic capacity, and glycolytic reversal were significantly increased compared with those in control-TuD-Muse cells (*p* < 0.001) (Fig. 6F), suggesting that let-7 originally suppressed ECAR. PI3Ki treatment reversed the enhanced ECAR in let-7a KD-Muse-cells (Fig. 6F). Glycolysis, glycolytic capacity, and glycolytic reversal were all significantly suppressed by PI3Ki (*p* < 0.001) compared with that in DMSO-treated let-7a-KD-Muse cells. At the same time, MEKi treatment did not have such an effect (Fig. 6F). Therefore, let-7 was suggested to inhibit glycolysis through the PI3K pathway, but not through the MEK/ERK pathway.

## Discussion

**Fig. 7:**
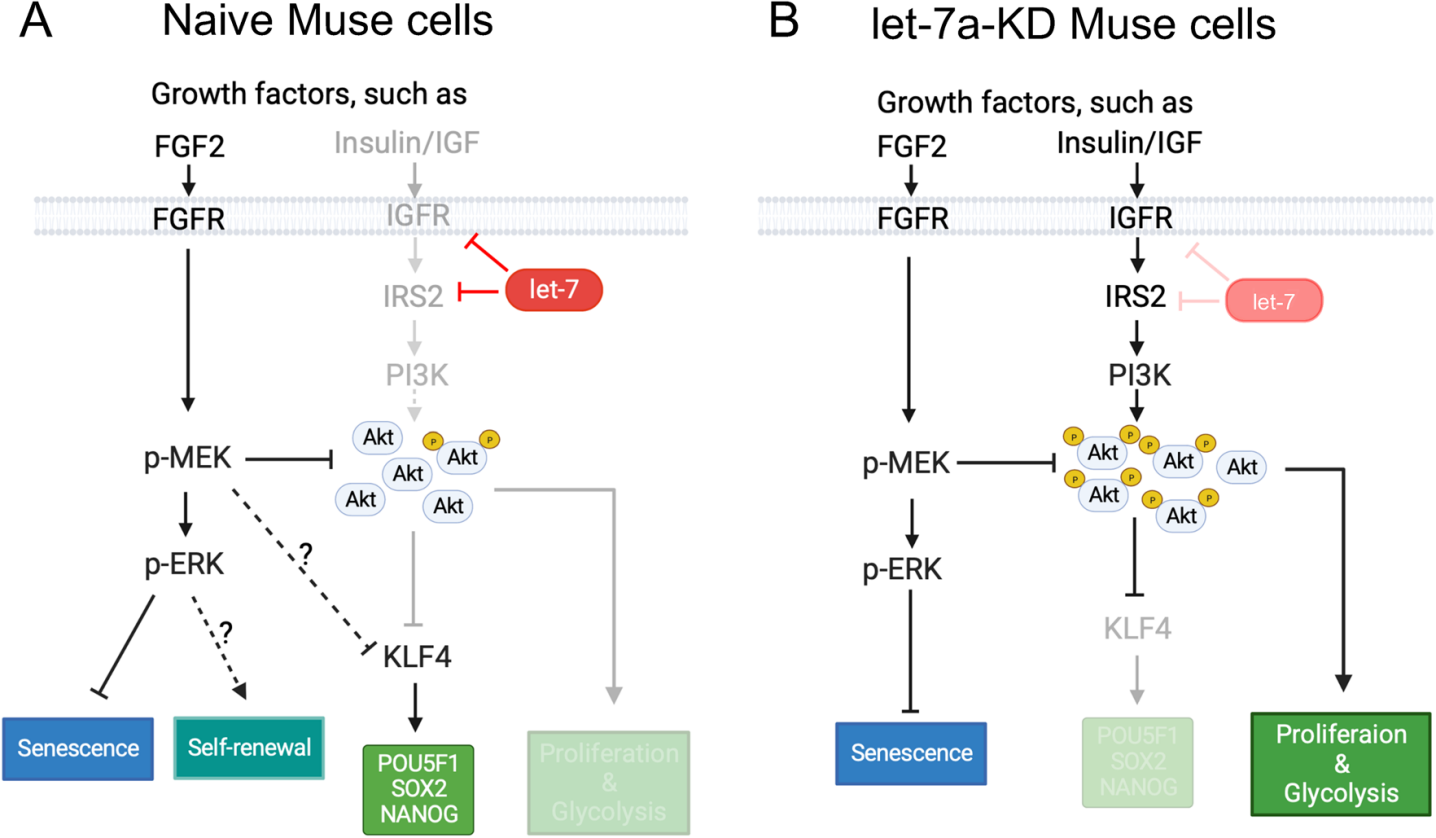
Summary. **A** The effect of let-7 on the PI3K-AKT and MEK/ERK pathways in naïve Muse cells. In naïve Muse cells, let-7 maintains the expression of pluripotency genes and inhibits proliferation and glycolysis. Let-7 inhibits the expression of IGF1R and IRS2 to repress the PI3K-AKT pathway. The PI3K-AKT pathway negatively controls the expression of *KLF4*, which promotes the expression of *POU5F1*, *SOX2*, and *NANOG*. The PI3K-AKT pathway directly inhibits the proliferation and glycolysis of naïve Muse cells. The other pathway, the MEK/ERK pathway, seems not affected by let-7. In naïve Muse cells, the MEK/ERK pathway suppresses the PI3K-AKT pathway by reducing the phosphorylation level of AKT. In addition, the MEK/ERK pathway also inhibits the expression of *KLF4*, but it virtually does not affect the expression of pluripotency genes. The MEK/ERK pathway is suggested to suppress senescence and maintain self-renewal of naïve Muse cells. **B** The effect of let-7 KD on the PI3K-AKT and MEK/ERK pathways in let-7-KD Muse cells. The PI3K-AKT pathway is more activated in let-7-KD-Muse cells than in naïve Muse cells. The increased AKT phosphorylation inhibits the expression of *KLF4*, which leads to the downregulation of the pluripotency genes *POU5F1*, *SOX2*, and *NANOG*. Cell proliferation and glycolysis were promoted. The MEK/ERK pathway reduces the phosphorylation level of AKT. The MEK/ERK pathway also suppresses cell senescence

In this study, we demonstrated a novel role of the tumor suppressor miRNA let-7 as a key player in maintaining pluripotency gene expression and repressing cell proliferation and glycolysis by inhibiting the PI3K-AKT pathway in endogenous pluripotent-like non-tumorigenic Muse cells. We also highlighted the importance of the MEK/ERK pathway in maintaining the Muse cell population among MSCs and suppressing their senescence. Different from ESCs and iPSCs, the system that depends on the tumor suppressor let-7 rather than oncogenic LIN28 to maintain pluripotent gene expression provides a low-risk model that enables both pluripotency and non-tumorigenicity in Muse cells. A summary of the proposed schema is shown in Fig. 7.

One outstanding difference between Muse cells and ESCs is the function of the PI3K-AKT pathway. In mouse ESCs, knockout of PTEN, a negative regulator of the PI3K-AKT pathway, upregulates KLF4, POU5F1, and NANOG levels [54]. In human ESCs, PI3K inhibition leads to decreased pluripotency gene expression [55, 56]. Thus, the PI3K-AKT pathway positively regulates pluripotency gene expression in ESCs. In Muse cells, however, we found that let-7 inhibited the expression of IGF1R and IRS2, leading to the suppression of PI3K-AKT downstream of IGF1R and IRS2, and sustained *KLF4* expression (Figs. 3, 4, 5). Consistently, PI3K-AKT inhibited the expression of *KLF4,* the factor upstream of *NANOG*, *POU5F1*, and *SOX2*; in naïve Muse cells*, KLF4, NANOG*, *POU5F1*, and *SOX2* were upregulated by PI3Ki treatment (Fig. 5C), and let-7a KD induced downregulation of these factors (Fig. 5D), which was counteracted by PI3Ki (Fig. 5F). These findings indicate that the PI3K-AKT pathway negatively regulates the expression of pluripotency genes in Muse cells, and that the inhibitory function of let-7 against PI3K-AKT is important for maintaining the expression of pluripotency genes.

We also found that KLF4 is a key regulator for sustaining pluripotency gene expression in Muse cells, as indicated by the suppression of *POU5F1*, *SOX2*, and *NANOG* after KLF4-siRNA introduction (Fig. 5H). In ESCs, KLF4 is downstream of the JAK/STAT3 pathway [49] and mediates the recruitment of Cohesin to the enhancer of *Pou5f1* to regulate *Pou5f1* transcription [57]. Furthermore, KLF4 directly binds to the *Nanog* promoter to drive its expression [50]. In this manner, KLF4 is able to act as the key regulator of pluripotency gene expression. Further studies are needed to determine how the PI3K-AKT pathway inhibits *KLF4* expression in Muse cells. The PI3K-AKT pathway is reported to activate the mammalian target of rapamycin complex 1 (mTORC1), leading to the inhibition of transcription factor E74-like factor 4, known to directly activate the expression of *KLF4* in T cells [58]. A similar mechanism might be functioning in Muse cells, but more detailed studies are required to elucidate whether or not the PI3K-AKT-mTORC pathway mediates KLF4 expression.

Let-7 is suggested to participate in the suppression of proliferation and glycolysis in Muse cells through inhibiting the IGF1R-IRS2-PI3K-AKT pathway (Fig. 6). Some cell cycle-relevant factors, such as cyclin D1, cyclin D2, CDK4, CDK6, and cell division cycle 25A, are reported to be let-7 targets [17, 59, 60]. Therefore, let-7 could suppress cell cycle activity by directly inhibiting those factors. In our microarray analysis, some of the cell cycle-relevant factors were upregulated in let-7 KD Muse cells (Fig. S2E), which might be the reason why let-7 KD accelerated proliferation. Glucose transporter type 4 increases glucose uptake under regulation of the PI3K-AKT pathway [61, 62]. By inhibiting the PI3K-AKT pathway, let-7 may inhibit glycolysis indirectly. In T cells, let-7 suppresses glycolysis by directly targeting hexokinase 2 mRNA, a critical enzyme in glycolysis [63]. Thus, it is possible that let-7 inhibits glycolysis in a direct or indirect manner in Muse cells. Further studies are needed to elucidate the detailed mechanisms. The telomerase activity was reported to be lower in Muse cells than that in HeLa cells and iPSCs [27, 29, 64]. Let-7a KD stimulated the proliferation but did not increase the telomerase activity, suggesting that let-7 is a key factor for controlling cell cycle but is not directly connected to tumorigenic proliferation in Muse cells.

Overactivation of the PI3K-AKT pathway based on genetic alterations of factors such as PIK3CA, PTEN, and AKT is frequently observed in various types of tumors [65]. After birth, the role of stem cells gradually changes from the rapid proliferation phase in the early stage of embryonic development to a moderate/slow proliferation phase in adult tissue where stem cells are required for tissue repair and cell replenishment [66]. Inhibition of the PI3K-AKT pathway plays a pivotal role in maintaining the quiescent state of somatic stem cells, such as muscle stem cells and hematopoietic stem cells [67–69]. High proliferation levels seem unfavorable for somatic stem cells to maintain a quiescent state. In ESCs, iPSCs, and cancer cells, high proliferation tends to accompany glycolytic metabolism, even in an oxygen-sufficient environment, to produce biomass [70]. In Muse cells, let-7 may be a barrier that prevents cells from unlimited proliferative activity.

The MEK/ERK pathway is suggested to participate in controlling cell cycle and proliferative activity through supporting self-renewal and suppressing senescence in Muse cells (Fig. 6 and S5), while not being directly involved in pluripotency gene expression (Fig. 5). The MEK/ERK pathway exhibits opposite effects between naïve and primed ESCs: Inhibition of ERK facilitates maintenance of the naïve pluripotent state, while on the other hand, activation of the MEK/ERK pathway by FGF2 maintains the primed pluripotent state (Weinberger et al., 2016). In Muse cells, FGF2 withdrawal (Fig. 1A) and/or MEK/ERK pathway inhibition (Fig. 5A) decreased the percentage of Muse cells among BM-MSCs, suggesting that the FGF2-MEK/ERK pathway may play a role in maintaining self-renewal of Muse cells. The MEK/ERK pathway, however, seems to have a limited effect on the expression of pluripotency genes in Muse cells (Fig. 5C). We also analyzed the possibility that the MEK/ERK pathway inhibits senescence. Senescent cells can be characterized by a flattened morphology, cell cycle arrest, and a β-Gal(+)/pRPS6(+)/Ki67(-) expression pattern [51, 52, 71]. Both MEKi-treated control-TuD and let-7-KD Muse cells exhibited a flattened morphology (Fig. 5B and E), growth arrest (Fig. 6C), and an increased ratio of β-Gal(+)/pRPS6(+)/Ki67(−) cells (Supplemental Fig. S5A and Fig. 6D). These findings suggest that the MEK/ERK pathway may play a role in preventing the senescence of Muse cells.

There are limitations to this study. Firstly, in this study, we found that MEK inhibited p-AKT (Fig. 4E), and p-AKT inhibited the expression of *KLF4* (Fig. 5C) in naïve Muse cells. Given these findings, inhibition of MEK was originally proposed to decrease the expression of *KLF4* via the activation of the PI3K-AKT pathway. However, our result showed that the inhibition of MEK upregulated the expression of *KLF4* in naïve Muse cells (Fig. 5C). Thus, MEK may regulate the expression of *KLF4* in Muse cells by an unknown PI3K-AKT-independent mechanism. The mechanism of how MEK regulates the expression of *KLF4* needs to be clarified in the future. Secondly, KLF4 was suggested to locate upstream of the three pluripotency genes by the KLF4 knockdown experiment (Fig. 5H). However, the upregulated *KLF4* expression induced by MEKi did not increase the expression of POU5F1, SOX2, and NANOG (Fig. 5C). This may be explained by the potential cell cycle arrest of Muse cells after MEKi treatment (Fig. 6C and Fig. S5) since cell cycle arrest was previously reported to decrease the expression of pluripotency genes [72]. The detailed mechanism should be clarified in the future. Thirdly, Muse cells could overexpress pre-let-7a-3 but not mature let-7a. On the other hand, NTERA2 could overexpress both pre-let-7a-3 and mature let-7a. This suggested that the lentiviral overexpression system worked in NTERA2 but not in Muse cells. Muse cells may have a unique defense mechanism to prevent overexpression of let-7, which remains to be elucidated.

## Materials and methods

### Cell culture

Human mesenchymal stem cells (hMSCs, LONZA, PT-2501), normal human dermal fibroblasts (NHDFs, LONZA, CC-2511), HEK293T, NTERA2, HeLa cells, and induced pluripotent stem cells (iPSCs) were used in this research. hMSCs and NHDFs were maintained in Minimum Essential Medium Eagle (αMEM, MilliporeSigma, M4526) supplemented with 10% fetal bovine serum (Hyclone, SH30910.03), 1 × GlutaMAX (Gibco, Thermo Fisher Scientific, 35050–061), 1 ng/mL human basic FGF2 (Miltenyi Biotech, 130–093-840) and kanamycin (Gibco, 15160–054). Culture medium was exchanged every 2 days. FGF2 was kept at 4 °C for no longer than 1 week and was freshly added while preparing the growth medium. HEK293T, NTERA2, and HeLa cells were maintained in Dulbecco’s modified Eagle’s medium (Gibco, 11965–092) supplemented with 10% fetal bovine serum, 1 mM sodium pyruvate (Gibco, 11360–070), and kanamycin. iPSCs were induced from NHDFs as previously described [29] and maintained in StemFit AK02N (AJINOMOTO, RCAK02N). The inhibitors used in this study were LY294002 (Selleck, S1105), PD0325901 (Wako, 162–25291), and MK2206 (Selleck, S1078). All the cells were cultured in a humidified incubator with 5% CO_2_ at 37 °C.

### Muse cell sorting

Muse cells were sorted when hMSCs or NHDFs reached 100% confluency. For antibody labeling, the cells were incubated with anti-SSEA-3 rat IgM antibody (1:1000, BioLegend, 330302) at 4 °C for 1 h following incubation with fluorescein (FITC) AffiniPure goat anti-rat IgM (1:100, Jackson ImmunoResearch, 112-095-075) or allophycocyanin-conjugated (APC) AffiniPure F (ab')_2_ fragment goat anti-Rat IgM (1:100, Jackson ImmunoResearch, 112-136-075) at 4 °C for 1 h. Purified Rat IgM, κ Isotype Ctrl Antibody (BioLegend, 400801) was used as a negative control for gate setting. FACS buffer (5% bovine serum albumin [BSA], 2 mM EDTA, and FluoroBrite Dulbecco’s modified Eagle’s medium [Thermo Fisher Scientific, A1896701]) was used for diluting the antibodies. Muse cells were collected using a BD FACSAria II SORP Flow Cytometer Cell Sorter (Becton Dickinson) in purify mode.

### Reverse transcription PCR (RT-PCR)

Total RNA was isolated with a mirVana™ miRNA Isolation Kit (Invitrogen, Thermo Fisher Scientific, AM1560) following the manufacturer's instructions. The quality and concentration of the total RNA were measured with a Nanodrop 1000 spectrophotometer (Thermo Fisher Scientific). cDNAs were generated from mRNAs by RT-PCR with a SuperScript III first-strand synthesis system (Invitrogen, 18080044) and oligo(dT)20 primers (Invitrogen, 18418020) using a Takara Thermal PCR Cycler (Takara Bio). RT-PCR of miRNAs was performed with a Taqman microRNA Reverse Transcription Kit (Invitrogen, 4366597).

### Quantitative PCR (qPCR)

qPCR was performed with either Taqman Universal Master Mix II, using UNG (Applied Biosystems, 4440038) or PowerUP SYBR Green Master Mix (Applied Biosystems, A25742) running in the 7500 Fast Real-Time PCR System (Applied Biosystems). When performing SYBR Green qPCR assays, we confirmed the melting curve to ensure the specificity of the PCR products. ACTB and RNU48 were used for mRNA and miRNA qPCR as endogenous controls, respectively. The 2^−ΔΔCT^ relative quantification method was used in all analyses for calculation. Primers used in this study were obtained in 3 ways: primer BLAST service was provided by the National Center for Biotechnology Information (NCBI), ordering of Taqman qPCR probes, and primer searches on Primer Bank (https://pga.mgh.harvard.edu/primerbank/). Supplemental Table 1 shows the details of the primers.

### Droplet digital PCR (ddPCR)

*Gene expression assay* Total RNA and cDNA were extracted and synthesized as described above. Taqman gene expression probes of LIN28A/B were used (Supplemental Table 1). cDNA samples and Droplet Generator Oil for Probe (Bio-Rad, 186-3005) were loaded in a DG8 cartridge (Bio-Rad, 186-4008), respectively. The QX200 Droplet Generator (Bio-Rad) was used to mix the samples and oil to generate the droplets. The mixed droplets were transferred to a 96-well twin.tec PCR Plate (Eppendorf, 95579) before sealing the plate with Foil Heat Seal (Bio-Rad, 1814040) in a PX1 PCR Plate Sealer (Bio-Rad). PCR was carried out on a C1000 Touch Thermal Cycler (Bio-Rad) at the following temperature and time: denaturation 95 °C, 30 s; annealing/extension 60 °C, 1 min for 40 cycles. The results were read in a Droplet Reader (Bio-Rad) and analyzed by QuantaSoft (Bio-Rad).

*ddTRAP* Telomerase activity was measured by ddTRAP following previously published protocol [73]. A whole cell lysate was prepared by adding NP40 lysis buffer. Telomerase extension (TS) primer (5′-AATCCGTCGAGCAGAGTT) was used in the extension reaction (25 °C, 1 h; 95 °C, min; 12 °C hold). In the extension reaction, no template control was used as a negative control. TS primer and ACX (reverse amplification) primer (5′-GCGCGGCTTACCCTTACCCTTACCCTAACC) were used to amplify the telomerase-extended substrates (95 °C, 30 s; 54 °C, 30 s; 72 °C, 30 s for 40 cycles). Droplet Generation Oil of EvaGreen (Bio-Rad, 186-4006) was used. The results were read in a Droplet Reader (Bio-Rad) and analyzed by QuantaSoft (Bio-Rad).

### Western blotting

Cells were washed with 1 × cold PBS twice and lysed by adding RIPA Lysis and Extraction Buffer (Thermo Fisher Scientific, 89900). A protease inhibitor (Thermo Fisher Scientific, 87785) and phosphatase inhibitor (Roche, 04906837001) were added to inhibit the protease and phosphatase activity. Cell lysates were centrifuged at 13,000 rpm at 4 °C for 10 min. The supernatant was transferred to new tubes, and protein quantification was performed using BCA Protein Assay Kit (Thermo Fisher Scientific, 23225) following the manufacturer's instructions. Sodium dodecyl sulfate–polyacrylamide gel electrophoresis (SDS-PAGE) was carried out with SDS-PAGE gels, and proteins were transferred to polyvinylidene difluoride membranes (Millipore, IPVH00010). Membranes were blocked in 5% skim milk (Nacalai, 31149–75)/Tris-buffered saline with 0.05% Tween-20 (TBST) for 1 h and incubated with primary antibodies overnight at 4 °C. Secondary antibody reactions were performed at room temperature for 1 h. The blots were washed 3 times with TBST at room temperature for 5 min after the primary or secondary antibody reactions. The blots were developed by Pierce ECL Plus Western Blotting Substrate (Thermo Fisher Scientific, 32,132). Images were acquired using Fusion FX imaging systems (Vilber). Band intensity was quantified by ImageJ software [74]. The primary and secondary antibodies used in this research are listed in Supplemental Table 2.

The following antibody dilutions were used: LIN28A (1:1000, Cell Signaling), LIN28B (1:1000, Cell Singling), IGF1 receptor β (1:1000, Cell Signaling), Insulin receptor β (1:1000, Cell Signaling), NRAS (1:200, Santa Cruz Biotechnology), p-AKT (T308) (1:1000, Cell Signaling), p-AKT (S473; 1:1000, Cell Signaling), AKT (1:1000, Cell Signaling), Phospho-p44/p42 MEK/ERK (ERK1/2; Thr202/Tyr 204) (1:1000, Cell Signaling), p44/p42 MEK/ERK (ERK1/2; 1:1000, Cell Signaling), β-actin (1:10,000, Abcam), and KLF4 (1:1000, Cell Signaling).

### Let-7 knockdown

pWPXL was a gift from Professor Didier Trono (Addgene #12257). Each pWPXL-U6-TuD-EF1α-GFP (or mCherry) of let-7a, -7b, -7e, and -7i was constructed. Lentivirus was created by the transduction of each plasmid with Lipofectamine 3000 (Invitrogen, L3000075) into HEK293T cells. Lentivirus was collected and concentrated using Ambion Ultra-15 centrifugal filters (Millipore, UFC910024) 72 h after transduction. The hMSCs were transfected with each lentivirus for 3 days. To allow for adequate collection of Muse cells after lentivirus infection, hMSCs were also cultured for 3–4 PDLs. TuD-let-7-transfected hMSCs were then harvested and labeled with anti-SSEA-3 antibody and either FITC or APC secondary antibody for sorting using the BD FACSAria II SORP Flow Cytometer Cell Sorter (Becton Dickinson).

### Luciferase assay

pGL4.13 (Promega, E6681) and pRL-CMV (Promega, E226A) are commercially available. The plasmid was first cut by XbaI and HindIII. A restriction enzyme BssHII site was then added artificially. The let-7 complementary sequences and ath-mir-416 complementary sequence yielded by annealing specific oligo sets were inserted into the restriction enzyme sites BssHII and XbaI of pGL4.13 to obtain pFluc-let-7a, pFluc-let-7b, pFluc-let-7e, and pFluc-let-7i plasmids. Let-7a, -7b, 7e, and -7i KD hMSC-Muse cells were sorted and plated on 12-well plates at a density of 15,000 cells/cm^2^. The modified pGL4.13 plasmids and pRL-CMV plasmid were co-transfected with Lipofectamine 2000 (Invitrogen) at a mass ratio of 30:1. Naive, control-TuD, and each type of let-7-KD-Muse cell were all transfected by pFluc-let-7a, pFluc-let-7b, pFluc-let-7e, and pFluc-let-7i plasmids (Fig. S2B). Luciferase assay was performed after 24 h using the Dual-Luciferase Reporter Assay System (Promega, E1910).

### Microarray and microarray analysis

Naïve, TuD-control-, let-7a-KD-, let-7b-KD-, let-7e-, and let-7i-KD-Muse cells were collected from hMSCs. Total RNA extraction, cDNA synthesis and hybridization, and microarray analysis were completed by Takara Bio Inc. The SurePrint G3 Human GE v3 8 × 60 K microarray (Agilent Technologies) was used for hybridization. Slides were scanned on the Agilent SureScan Microarray Scanner (G2600D) using the 1-color scan setting. The scanned images were analyzed by Feature Extraction Software (Agilent Technologies).

*Normalization* For calculating the scaling factor, a trimmed mean probe intensity was settled by removing 2% of the lower and higher end of the probe intensities. Using the scaling factor, the normalized signal intensities were calculated.

*Showing differential expression by heatmap* Genes whose expression was under detection limit were filtered in Microsoft Excel, and the heatmap of differential expression was produced by the pheatmap package of R (https://CRAN.R-project.org/package=pheatmap).

### Cell starvation and growth factor treatment

Muse cells were seeded at 15,000 cells/cm^2^. After attaching, medium was changed to serum-free αMEM, and cells were further cultured for 8 h. After the addition of 100 ng/mL insulin, 100 ng/mL IGF1, and 2 ng/mL FGF2 to the corresponding wells, the cells were treated for 15 min. Cells were then lysed, and the samples were analyzed by Western blotting.

### Flow cytometry analysis of the ratio of the Muse population

Cells were seeded at 1 million/10 cm dish. After adherence, medium was changed to culture medium with FGF2, without FGF2, containing 5 µM LY294002, and containing 1 μM PD0325901, respectively. After culturing for 2 PDLs, cells were then collected and stained with anti-SSEA-3 antibody as described above. The positive ratio of SSEA-3 + cells was analyzed by the CytoFLEX S Flow Cytometer (Beckman Coulter). Flow cytometry data were analyzed by Kaluza Analysis Software (Beckman Coulter).

### Analysis of pluripotency gene expression

Naive and let-7-KD Muse cells were each collected and seeded at 15,000 cells/cm^2^. After attaching, cells were treated with DMSO, 5 µM LY294002, or 1 µM PD0325901, respectively, for 24 h. Gene expression was analyzed by qPCR as described above.

### Let-7 overexpression

The let-7 overexpression plasmids are commercially available from System Biosciences (SBI). The catalog numbers of the plasmids used in this research are PMIRH000PA-1 (empty vector), PMIRHlet7a3PA-1 (let-7a overexpression), PMIRHlet7bPA-1 (let-7b overexpression), PMIRHlet7ePA-1 (let-7e overexpression), and PMIRHlet7iPA-1 (let-7i overexpression). Lentivirus was created by transduction of each plasmid with Lipofectamine 3000 (Invitrogen, L3000075) into HEK293T cells. Lentivirus was collected and concentrated by Ambion Ultra-15 centrifugal filters (Millipore, UFC910024) 72 h after transduction. The hMSCs were transfected by each lentivirus for 3 days. To obtain enough number of Muse cells after lentivirus infection, hMSCs were also cultured for 3–4 PDLs. TuD-let-7-transfected hMSCs were then harvested and labeled with anti-SSEA-3 antibody and APC secondary antibody for sorting using the BD FACSAria II SORP Flow Cytometer Cell Sorter (Becton Dickinson).

### siRNA knockdown

Three kinds of Klf4 siRNA (Ambion, Thermo Fisher Scientific, ID: S17793, S17794, and S17795) were mixed up, and naive Muse cells were sorted and transfected with 25 pmol KLF4 siRNA or 25 pmol scrambled siRNA (Ambion) by Lipofectamine RNAiMAX (Invitrogen, 13778–150) in 6-well plates for 48 h following the manufacturer’s instructions. Total RNA was extracted, and gene expression was analyzed by qPCR as described above.

### Cell cycle analysis

Naive, control-TuD, let-7-KD-Muse cells were collected, respectively. 70% ethanol was pre-cooled at − 30 °C. Fix 120,000 cells from each group just after sorting with pre-cooled 70% ethanol at 4 °C for 30 min. The left cells were seeded as 25,000 cells/cm^2^ in 12 wells. Cells were detached from the culture plates every 12 h and then fixed with pre-cooled 70% ethanol. After fixation, all the samples were stored at 4 °C. Centrifuge all the samples at 1000 × g for 5 min at room temperature and wash with FACS buffer. Centrifuge again at 1000 × g for 5 min at room temperature. Digest cells with 250 µg/mL RNase at 37 °C for 15 min. Add Propidium Iodide (PI) to each sample and incubate at 4 °C for 10 min. Analyze the samples by CytoFLEX S Flow Cytometer (Beckman Coulter). All the data were analyzed by Kaluza Analysis Software (Beckman Coulter).

### Cell proliferation assay

Cells were seeded in 4-well plates at 5000 cells/well. Cells were trypsinized and counted by hemacytometers (WakenBtech, WC2-100) every 24 h until day 6.

### ECAR analysis

We seeded 10,000 cells per well in Seahorse XF96 cell culture microplates (Agilent Technologies, 103725–100) and pre-cultured cells 12 h before measurement. ECAR was then measured using the Seahorse XFe96 Analyzer (Agilent Technologies). We calculated fundamental parameters such as glycolysis, glycolytic capacity, and glycolytic reverse of ECAR following the manufacturer's instructions.

### Flow cytometry analysis of apoptosis

*TUNEL assay* Muse cells were collected and fixed with fresh 4% paraformaldehyde in PBS, pH 7.4 for 30 min, further incubated with permeabilization solution (0.1% Triton × 100 in 0.1% sodium citrate solution) for 2 min on ice, and labeled by the DeadEnd Fluorometric TUNEL System (Promega, G3250) following the manufacturer's instructions. A positive control was prepared with a DNase-digested sample. Apoptosis of Muse cells was observed by flow cytometry using a BD FACSAria II SORP Flow Cytometer Cell Sorter.

*Annexin V staining* hMSCs treated with UV for 5 min followed by incubation for an additional 24 h were used as a positive control. Muse cells were seeded 20,000 cells/cm^2^ in a 24-well-plate and the medium was changed to DMSO with the addition of 5 μM LY294002 or 1 μM PD0325901 after cell attachment. Cells were collected 1, 3, and 5 days after inhibitor treatment and stained using a MEBCYTO Apoptosis Kit (Annexin V-FITC Kit; MBL, 4700) following the manufacturer’s instruction. Samples were analyzed by the CytoFLEX S Flow Cytometer (Beckman Coulter). Flow cytometry data were analyzed using Kaluza Analysis Software (Beckman Coulter).

### Flow cytometry analysis for senescence

Cells were stained with the SPiDER-βGal detection kit (Dojindo, SG02) following the manufacturer's protocol and then collected by trypsinization. The mean fluorescence intensity (MFI) was analyzed by CytoFLEX S Flow cytometer (Beckman Coulter). The calculation method is shown below:

Sample A (experimental group): Cells stained with SPiDER-βGal.

Sample B (experimental group): Cells without SPiDER-βGal staining.

Sample C (control group): Cells stained with SPiDER-βGal.

Sample D (control group): Cells without SPiDER-βGal staining.

Variation of SA-β-gal activity = (MFI of A − MFI of B) − (MFI of C − MFI of D).

### Immunofluorescent staining for senescent cells

Control-TuD and let-7a KD Muse cells were sorted and seeded at a density of 6,600 cells per 96-well (22,000 cells/cm^2^). Subsequently, they were treated with either DMSO or 1 µM PD0325901 for a duration of 5 days. Following treatment, the cells were washed once with 1 × PBS and fixed with 2% Paraformaldehyde (PFA)/PBS for 5 min at room temperature. After three washes with 1 × PBS, SA-βgal staining was performed using a staining solution for 12 h at 37 °C. After SA-βgal staining, cells were permeabilized with ice-cold 100% methanol at 4 °C for 15 min. Then, cells were treated with 0.4 M peroxide for 5 min at room temperature to quench the mCherry signal. This signal had been introduced into Muse cells through lentivirus, serving as an indicator for FACS sorting of control-TuD and let-7a KD Muse cells, as mentioned above. Cells were washed three times with 1 × PBS and blocked with a blocking solution for 1 h at room temperature before overnight incubation with the primary antibody Ki67 at 4 °C. The following day, cells were washed three times with 1 × PBS and incubated with the Alexa Fluor 647 secondary antibody at room temperature for 1 h. Subsequently, cells were stained with the another primary antibody pRPS6 for 1 h, followed by incubation with the Alexa Fluor 488 secondary antibody for another 1 h. Finally, cells were stained with DAPI for 5 min. After 3 washes with 1 × PBS, the stained cells were imaged with a fluorescence microscope (Keyence, BZ-X710).

Solutions and antibody dilutions were as follows: SA-βGal staining solution (400 mM Potassium Ferricyanide, 400 mM Potassium Ferrocyanide, 1 mg/mL X-gal, 2 mM MgCl_2_, and 30 mM NaCl in citrate/phosphate buffer, pH = 6.0); blocking solution (20% Blockace (KAC, UKB40), 5% BSA, 0.3% Triton X-100 in PBS); primary antibody dilution buffer (5% Blockace, 1% BSA, 0.3% Triton X-100 in PBS); secondary antibody dilution buffer (0.2% Triton X-100 in PBS). Ki67 primary antibody (1:250, Abcam), pRPS6 primary antibody (1:400, Cell signaling), Alexa Fluor 647-conjugated AffiniPure F(ab’)_2_ Fragment Donkey Anti-Rabbit IgG (H + L) (1:200, Jackson ImmunoResearch), and Alexa Fluor 488-conjugated AffiniPure F(ab')_2_ Fragment Donkey Anti-Mouse IgG (H + L) (1:200, Jackson ImmunoResearch).

### Statistical analysis

All statistical analyses were performed in Microsoft Excel or Graphpad Prism 8.0. Bioinformatic analysis was performed in R programming (Version 3.5.1). Data are presented as mean ± SD. The statistical significance of differences between 2 groups was calculated by the unpaired Student’s t-test. For comparison of more than 2 groups, 1-way ANOVA was conducted with Tukey’s post-hoc test. (*p < 0.05, **p < 0.01, ***p < 0.001, ns: no significance).

### Supplementary Information

Below is the link to the electronic supplementary material.

Supplementary file1 (DOCX 7450 KB)

## Data Availability

All the data is contained within the article and supporting information and will be available upon request.
