# Supplementary material for: Tumor suppressor let-7 acts as a key regulator for pluripotency gene expression in Muse cells
Source: Cell Mol Life Sci. 2024 Jan 23;81(1):54. doi: 10.1007/s00018-023-05089-9 (PMC10805825; doi:10.1007/s00018-023-05089-9)
Supplement: Supplementary file 1 — Supplementary file1 (DOCX 7450 KB) [file 18_2023_5089_MOESM1_ESM.docx]

**Supplemental Figure S1. NHDF-Muse cells express let-7 but not LIN28.**

(A-B) FACS sorting of Muse cells.

1. qPCR showing the expression of *LIN28A/B* in NHDF-Muse cells. ACTB was used as an endogenous control.
2. Western blot showing LIN28A/B levels in NHDF-Muse cells, NTERA2, and iPSCs. β-Actin was used as an endogenous control.
3. Expression of let-7 subtypes in NHDF-Muse cells (all n=3). RNU48 was used as an endogenous control.
4. qPCR showing the expression of let-7a, -7b, -7e, and -7i in Muse cells and iPSCs (n=3). RNU48 was used as an endogenous control. A log10 scale was used for the y-axis.

**Supplemental Figure S2. Let-7 knockdown.**

1. Structure of TuD-hsa-let-7a (left) and construction of the lentiviral vector for let-7 knockdown (right).
2. Scheme of the luciferase assay to check the knockdown effect of TuD-let-7. x in let-7x represents a, b, e, or i.
3. Sequence comparisons of hsa-let-7a-5p, hsa-let-7b-5p, hsa-let-7e-5p, and hsa-let-7i-5p.
4. Examination of cellular apoptosis by TUNEL assay. Ctrl: control
5. KEGG pathway analysis of the cell cycle.

**Supplemental Figure S3. Analysis of senescence and apoptosis.**

(A-B) Bar graph showing the analysis of flow cytometric detection of SA-βgal-expressed cells in control-TuD and let-7a-KD Muse cells (All n=3).

1. Annexin-V-FITC staining for detecting cellular apoptosis after PI3Ki and MEKi treatment.
2. Western blot showing the knockdown effect of KLF4 siRNA. β-Actin was used as an endogenous control. The KLF4 expression intensity was normalized by β-actin.

PI3Ki: LY294002. MEKi: PD0325901.

**Supplemental Figure S4. Let-7 overexpression in Muse cells.**

1. Design of the let-7 overexpression system. copGFP was used as an indicator of positively transfected cells.
2. Comparison of the expression of let-7a-1, let-7a-2, and let-7a-3 in Muse cells (n=3).
3. qPCR analysis showing the overexpression of pre-let-7a-3 and mature let-7 (n=3).
4. qPCR showing the overexpression of pre-let-7a-3 and mature let-7a in NTERA2 (both n=3). Empty vector-transfected cells were used as a negative control.
5. Luciferase assay to confirm the effect of let-7i overexpression (n=5). Renilla luciferase was used as a control.
6. qPCR analysis of the expression of pluripotency genes before and after let-7i overexpression (n=3).

OE: overexpression. ACTB and RNU48 were used as endogenous controls for gene expression and miRNA expression, respectively.

**Supplemental Figure S5. SA-βGal/pRPS6/Ki67 staining for senescent cell counting.**

(A) Staining for β-gal, pRPS6, and Ki67. DAPI was used for nuclear staining. Scale bar: 100 µm. MEKi: PD0325901

**Supplemental Table 1: Primers**

| **Name** | **Sequence (5'→3') or assay name** |  |
| --- | --- | --- |
| *LIN28A* | Taqman Gene Expression Assays | Hs00702808_s1 |
| *LIN28B* | Taqman Gene Expression Assays | Hs01013729_m1 |
| hsa-let-7a | Taqman MicroRNA Assays | Assay ID: 000377 |
| hsa-let-7b | Taqman MicroRNA Assays | Assay ID: 002619 |
| hsa-let-7c | Taqman MicroRNA Assays | Assay ID: 000379 |
| hsa-let-7d | Taqman MicroRNA Assays | Assay ID: 002283 |
| hsa-let-7e | Taqman MicroRNA Assays | Assay ID: 002406 |
| hsa-let-7f | Taqman MicroRNA Assays | Assay ID: 000382 |
| hsa-let-7g | Taqman MicroRNA Assays | Assay ID: 002282 |
| hsa-let-7i | Taqman MicroRNA Assays | Assay ID: 002221 |
| hsa-mir-98 | Taqman MicroRNA Assays | Assay ID: 000577 |
| RNU48 | Taqman MicroRNA Assays | Assay ID: 001006 |
| hsa-let-7a-1 | Taqman Pri-miRNA Assay | Hs03302533_pri |
| hsa-let-7a-2 | Taqman Pri-miRNA Assay | Hs03302539_pri |
| hsa-let-7a-3 | Taqman Pri-miRNA Assay | Hs03302546_pri |
| *ACTB* | Taqman Gene Expression Assays | Hs03023880_g1 |
| *ACTB*-F | CATGTACGTTGCTATCCAGGC | PrimerBank ID: 4501885a1 |
| *ACTB*-R | CTCCTTAATGTCACGCACGAT | PrimerBank ID: 4501885a1 |
| *POU5F1*-F | AACCCACACTGCAGCAGATCA | NCBI Primer-BLAST |
| *POU5F1*-R | ACACTCGGACCACATCCTTC | NCBI Primer-BLAST |
| *SOX2*-F | TCCAACATCCTGAACCTCAGC | NCBI Primer-BLAST |
| *SOX2*-R | TCTGCGTCACACCATTGCT | NCBI Primer-BLAST |
| *NANOG*-F | CAGCTCGCAGACCTACATGA | NCBI Primer-BLAST |
| *NANOG*-R | CTCGGACTTGACCACCGAAC | NCBI Primer-BLAST |
| *KLF4*-F | CACCCACACTTGTGATTACGC | NCBI Primer-BLAST |
| *KLF4*-R | TGTTTACGGTAGTGCCTGGTC | NCBI Primer-BLAST |

**Supplemental Table 2: Primary and Secondary antibodies**

| **Antibodies** | **Manufacturer** | **Catalog number** |
| --- | --- | --- |
| Purified anti-human/mouse SSEA-3 Antibody | BioLegend | 330302 |
| Purified Rat IgM, κ Isotype Ctrl Antibody | BioLegend | 400801 |
| Fluorescein (FITC) AffiniPure Goat Anti-Rat IgM, µ chain specific | Jackson ImmunoResearch | 112-095-075 |
| Allophycocyanin (APC) AffiniPure F(ab')₂ Fragment Goat Anti-Rat IgM, µ chain specific | Jackson ImmunoResearch | 112-136-075 |
| LIN28A | Cell Signaling | 3978S |
| LIN28B | Cell Signaling | 4196S |
| IGF-I Receptor β | Cell Signaling | 9750 |
| Insulin Receptor β | Cell Signaling | 23413 |
| IRS2 | Abcam | Ab134101 |
| NRAS | Santa Cruz Biotechnology | SC-31 |
| β-actin | Abcam | ab6276 |
| Phospho-AKT (T308) | Cell Signaling | 13038S |
| Phospho-AKT (S473) | Cell Signaling | 9271 |
| AKT | Cell Signaling | 9272 |
| Phospho-p44/p42 MEK/ERK (ERK1/2) (Thr202/Tyr 204) | Cell Signaling | 9101 |
| p44/p42 MEK/ERK (ERK1/2) | Cell Signaling | 9102 |
| KLF4 | Cell Signaling | 12173S |
| Peroxidase AffiniPure Goat Anti-Mouse IgG | Jackson ImmunoResearch | 115-035-071 |
| Peroxidase AffiniPure Goat Anti-Rabbit IgG (H+L) | Jackson ImmunoResearch | 111-035-144 |
| Ki67 | Abcam | Ab16667 |
| Alexa Fluor 488-conjugated AffiniPure F(ab')₂ Fragment Donkey Anti-Mouse IgG (H+L) | Jackson ImmunoResearch | 711-546-150 |
| Phospho-S6 Ribosomal Protein (Ser235/236) (E2R1O) Mouse Ab | Cell signaling | 62016S |
| Alexa Fluor 647-conjugated AffiniPure F(ab’)_2_ Fragment Donkey Anti-Rabbit IgG (H+L) | Jackson ImmunoResearch | 711-606-152 |
